# Cell-Permeable Microprotein from Panax Ginseng Protects Against Doxorubicin-Induced Oxidative Stress and Cardiotoxicity

**DOI:** 10.3390/antiox14040493

**Published:** 2025-04-19

**Authors:** Bamaprasad Dutta, Shining Loo, Antony Kam, Xiaoliang Wang, Na Wei, Kathy Qian Luo, Chuan-Fa Liu, James P. Tam

**Affiliations:** 1 School of Biological Sciences, Nanyang Technological University, 60 Nanyang Drive, Singapore 637551, Singapore; bama0001@e.ntu.edu.sg (B.D.); shining.loo@xjtlu.edu.cn (S.L.); antony.kam@xjtlu.edu.cn (A.K.); wangxl@imm.ac.cn (X.W.); cfliu@ntu.edu.sg (C.-F.L.); 2 School of Pharmacy, The Neotia University, Sarisa, Diamond Harbour Road, 24 Parganas (South), West Bengal 743368, India; 3 Wisdom Lake Academy of Pharmacy, Xi’an Jiaotong-Liverpool University, Suzhou 215123, China; 4 Department of Biological Sciences, Xi’an Jiaotong-Liverpool University, Suzhou 215123, China; 5 Institute of Materia Medica, Chinese Academy of Medical Sciences, Beijing 100050, China; 6 School of Chemistry, Chemical Engineering and Biotechnology, Nanyang Technological University, 70 Nanyang Drive, Singapore 637457, Singapore; na.wei@tu-darmstadt.de; 7 Faculty of Health Sciences, University of Macau, Taipa, Macao SAR, China; kluo@um.edu.mo; 8 Ministry of Education Frontiers Science Center for Precision Oncology, University of Macau, Taipa, Macao SAR, China

**Keywords:** ginsentide, cardioprotective adjuvant, doxorubicin-induced cardiotoxicity, oxidative stress, antioxidation, cysteine-rich peptide, microprotein

## Abstract

(1) Background: Doxorubicin (DOX) is a frontline chemotherapeutic, but its side-effects from oxidative stress, leading to cardiotoxicity, pose significant challenges to its clinical use. We recently discovered a novel family of proteolysis-resistant, cystine-dense, and cell-penetrating microproteins from *Panax ginseng* that we term ginsentides. Ginsentides, such as the 31-residue TP1, coordinate multiple biological systems to prevent vascular dysfunction and endoplasmic reticulum stress induced by internal and external stressors. (2) Methods: We assessed the protective effects of ginsentide TP1 on DOX-induced cardiotoxicity using both in vitro functional studies on H9c2 cardiomyocytes and in vivo animal models by zebrafish and ICR mouse models. In these models, we examined oxidative stress, apoptosis, intracellular calcium levels, mitochondrial function, inflammatory responses, and cardiac function. (3) Results: We show that ginsentide TP1 protects against DOX-induced cytotoxicity in the mitochondria-rich H9c2 cardiomyocytes and reduces myocardial injury in zebrafish and mice by mitigating oxidative stress, inflammation, calcium, and mitochondrial dysfunction, as well as apoptosis-mediated cell death. Importantly, TP1 preserves cellular homeostasis without compromising the anticancer potency of DOX in breast cancer cells. (4) Conclusions: our findings highlight a specific antioxidative function of ginsentide TP1 in managing DOX-induced cardiotoxicity during cancer treatment and provide a promising lead for developing cardioprotective peptides and microproteins against oxidative stress.

## 1. Introduction

For half a century, doxorubicin (DOX), which is a member of anthracycline, has been a commonly used chemotherapeutic agent for various cancers, including solid tumours such as breast carcinoma, bladder carcinoma, lung cancer, and soft tissue sarcoma, as well as lymphoma, leukaemia, and metastatic cancers [1,2,3,4]. At nanomolar concentrations, DOX induces a potent cytotoxic effect against multiple intracellular targets, leading to the immune-mediated clearance of cancer cells and making it the first chemotherapeutic drug of its kind [2]. However, DOX produces severe off-target, dose-dependent, and cumulative side-effects [3,4,5,6]. Cardiac damage attributed to the oxidative stress of mitochondria was observed in over 18% of adult patients receiving cumulative DOX doses over 450 mg/m^2^ [7,8]. This prevalence rose to 36% when the total cumulative dosage surpassed 600 mg/m^2^ over four decades [7,8]. In a study of children who received cumulative Dox doses surpassing 410 mg/m^2^, 32% exhibited left ventricular dysfunction [9]. Developing an effective treatment to counter the lingering long-term cardiotoxic effects of DOX-treated young adults remains an urgent challenge.

Attempts to reduce the cardiotoxicity by the slow administration of doxorubicin via prolonged infusion instead of bolus administration have reduced the anticancer efficacy while increasing the risk of metastasis [10,11]. The DOX analogues, including epirubicin and mitoxantrone, have fewer cardiotoxic side-effects, but also significantly reduced the chemotherapeutic efficacy [12]. Formulations like liposomal- and nanoparticle-based delivery systems are emerging approaches to managing dosage-dependent toxicity [13,14,15,16]. In addition, recent advances in targeted drug delivery and biologics have also facilitated tumour tissue-specific DOX accumulation that could improve the therapeutic efficacy and reduce side-effects [16]. However, higher dosages still pose a risk of cumulative dose-dependent cardiotoxicity.

Another approach to reducing DOX-related toxicity involves adjuvant therapies with cardioprotective agents like haematopoietic cytokines, miRNAs, and natural compounds [17,18,19,20,21,22], as well as free-radical inhibitors like dexrazoxane [18,19,20], N-acetylcysteine [22], reduced glutathione (GSH), coenzyme Q10, vitamin A, and α-tocopherol [17,21]. However, these inhibitors often compromise the therapeutic efficacy of DOX and potentially increase the susceptibility to secondary malignancies. Thus, a specific adjuvant that can reduce the risk of DOX-mediated cardiotoxicity without compromising its anticancer potential is highly desirable.

**Figure 1:**
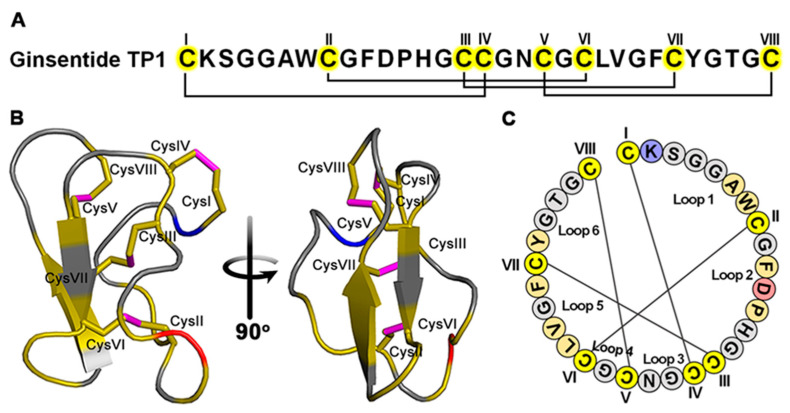
Ginsentide TP1 is a cystine-dense pseudocyclic peptide from ginseng. (**A**) Primary structure of ginsentide TP1 from *Panax ginseng* and *P. notoginseng*, showing disulphide linkages and its pseudocyclic structure with both of its terminals, C(I) and C(VIII), forming disulphides with internal cysteine. (**B**) The 3D structure of ginsentide TP1 (PDB: 2ML7) to support its compact and pseudocyclic structure. (**C**) Loops formed by four disulphides to highlight the pseudocyclic nature of TP1 with the N-terminal loop1 and the C-terminal loop6 in close proximity [see the top of TP1 3D structure in (**B**)]. Coloured circles indicate amino acid residues and connecting lines depicting disulphide bonds.

*Panax ginseng* is known for its “cure-all” effects, and its crude extracts are well documented as effective in treating cardiovascular diseases [23,24,25,26,27,28,29]. Recently, our laboratory identified peptidyl bioactive compounds from *Panax ginseng* which are primarily responsible for this “cure-all” effect. Contrary to conventional belief, these compounds, which we term ginsentides, are not small-molecule metabolites such as the well-studied ginsenosides [27,28], but belong to a novel cysteine-rich peptides (CRPs) family of microproteins [30]. Among the 14 identified ginsentides, TP1-14, the prototypic ginsentide TP1 is most abundant in the commercially popular ginseng species from China and America, *P. ginseng* and *P. notoginseng* [30]. TP1, a 31-amino acid peptide, contains eight cysteine residues forming four cross-linking disulphide bonds that create a highly compact, pseudocyclic structure with the first and last residues connected by a disulphide (Figure 1A–C) [30]. Consequently, the structurally defined and disulphide-constrained microprotein TP1 is resistant to enzymatic degradation by both exopeptidases and endopeptidases, enabling its oral bioavailability [30,31,32]. Ginsentides generally exhibit a broad-spectrum bioactivity characteristic of adaptogens, which primarily relieves stress [31,32,33]. In addition, ginsentides like TP1 and its homologs are also cell-penetrating, though many of their intracellular targets remain unexplored [31,32,33].

DOX and other anthracyclines bind to intracellular targets like topoisomerase-II, p53, and, importantly, cardiolipin in mitochondria, eliciting multiple modes of action. The primary mechanisms proposed for DOX-induced cardiotoxicity includes redox cycling and ROS generation [34,35,36,37], which results in oxidative stress, disruptions in the mitochondrial function, apoptosis, and calcium dysregulation. Recently, we demonstrated that TP1 maintains protein homeostasis and suppresses ER-stress-linked apoptosis in hypoxic cells through the eNOS/NO signalling pathways [31]. We envisioned that these TP1 functions could be exploited as an adjuvant therapy to prevent DOX-induced cardiotoxicity and cellular stress.

Here, we report the specific cardioprotective effect of TP1 against DOX-induced cardiotoxicity using both in vitro H9c2 rat cardiomyocytes and in vivo models, including zebrafish and mouse studies. We show that TP1 co-treatment with DOX reduces DOX-mediated oxidative stress. Importantly, ginsentide TP1 reduces mitochondrial and smooth endoplasmic reticulum (SER) dysfunction, preventing DOX-mediated apoptosis in cardiomyocytes. As such, TP1 protects against DOX-induced cardiotoxicity and restores cardiac function in DOX-treated zebrafish and mouse models. Collectively, this work provides new directions for developing specific adjuvant therapies to reduce DOX-induced toxicity in cancer treatments.

## 2. Materials and Methods

### 2.1. Reagents

Unless specified otherwise, chemicals and reagents were sourced from Sigma-Aldrich (St. Louis, MO, USA). Doxorubicin (Lot # C21PB55410B) was acquired from Saitong, China, and resveratrol (Lot # 58706) was obtained from MCE, China. Anti-GAPDH (6C5) primary antibody was purchased from Santa Cruz Biotechnology (Santa Cruz, CA, USA). Primary against cleaved caspase-3 (Asp175) and HRP-linked secondary antibodies including anti-mouse IgG and anti-rabbit IgG antibodies were acquired from Cell Signalling Technology (Danvers, MA, USA). We procured the ECL substrate (Clarity Max Western ECL Blotting Substrate, #1705062) from Bio-Rad Laboratories, Segrate, Italy. The cell lines used in our studies—H9c2 (ATCC CRL-1446), MDA-MB-231 (ATCC HTB-26), and MCF7 (ATCC HTB-22)—were obtained from the American Type Culture Collection (ATCC) in Manassas, VA, USA.

### 2.2. Isolation and Purification of Ginsentide TP1

Ginsentide was extracted from *P. ginseng* flowers using our previously reported protocol [30,31,32]. In brief, dried flowers were ground and mixed with Milli-Q water (100 g/L). The crude extract was further processed through centrifugation, followed by filtration using a 0.22 μm membrane (Thermo Fisher Scientific, Waltham, MA, USA) to obtain a clean supernatant. TP1 peptides were isolated and purified from the obtained extract through multiple rounds of reversed-phase high-performance liquid chromatography (RP-HPLC). The RP-HPLC was performed on a 5 μm C18 250 × 22 mm column (Phenomenex, Torrance, CA, USA) with a flow rate of 5 mL/min, employing a linear gradient increasing from 10–80% buffer B [0.1% trifluoroacetic acid (TFA) in acetonitrile (ACN)] at 1% per minute, while buffer A consisted of 0.1% TFA in HPLC-grade water. For the final purification step, peptide separation was performed using a smaller C18 250 × 4.6 mm column (5 μm, Phenomenex, Torrance, CA, USA) at a flow rate of 1 mL/min. A linear gradient of buffer B [0.1% trifluoroacetic acid (TFA) in acetonitrile (ACN)] was applied, increasing from 10–80% at rate of 1% per minute with buffer A [0.1% TFA in HPLC-grade water]. TP1-containing fractions were identified using MALDI-TOF mass spectrometry, lyophilised, and stored for further use. It should be noted that although ginsentide TP1 is found abundantly in ginseng roots and flowers, ginseng flowers are much less expensive than roots for our extraction purpose [30].

### 2.3. Culture of Cells and Subsequent Treatment

H9c2 rat cardiomyocytes and MCF7 human breast cancer cells were cultured and maintained in DMEM growth medium supplemented with 10% FBS, along with antibiotics (Penicillin 100 IU/mL and Streptomycin 100 µg/mL), under standard conditions of 37 °C and 5% CO_2_. Pre-cultured cells were subjected to either PBS as mock treatment or the respective drug treatments. IC_50_ of DOX to H9c2 cells was determined and 2 µM DOX was used for the rest of the experiments. TP1 activities were assessed using a 0–100 µM dose range, and 10 µM TP1 was considered for the rest of the experiments. Post-treated cells were then subjected to use in experimental assessment.

### 2.4. LDH Assay

Cytotoxicity and membranolysis were assessed using an LDH release-based cytotoxicity assay with LDH Cytotoxicity Assay Kit (CytoSelect™, #CBA-241, Cell Biolabs, Inc., San Diego, CA, USA). In short, the culture media from pre-treated cells were harvested, combined with the LDH assay reagent in a 9:1 ratio, and incubated for 2 h at 37 °C. Colorimetry-based quantification was conducted to measure the LDH release. The assay was performed in triplicate, with PBS serving as the mock treatment and 1% Triton X-100 employed as a positive control to induced cell death and disrupt the membrane.

### 2.5. MTT Assay

Pre-treated cells were exposed to MTT reagent with final concentration of 0.5 mg/mL and incubated at 37 °C for 2 h. The resulting formazan crystals were isolated by aspirating the culture medium and were quantified colorimetrically after being dissolved in dimethyl sulphoxide. The colorimetric analysis was carried out at 570 nm with a plate reader (Tecan Magellan, Männedorf, Switzerland), using 630 nm as the reference wavelength. Data were obtained from three separate experiments.

### 2.6. Western Blotting

Proteins were isolated from 24 h pre-treated cells. Then, 30 µg of protein from each protein sample was separated using a 10% acrylamide gel and subsequently electrotransfered onto a PVDF membrane. Immunoblotting was conducted with antibodies specific to the target protein and detected with ECL system.

### 2.7. Immunoassay Using ELISA

Cell lysates and culture medium were used to quantitatively assess the expression and secretion of TNF-α and IL-6 through enzyme-linked immunosorbent assay (ELISA). Briefly, H9c2 cells were plated in a 24-well plate at a density of 3 × 10^5^ cells, cultured, and subsequently treated with respective chemicals followed by collection and processing of the cell pellet and culture media, as per the manufacturer’s protocol. Rat Interleukin-6 ELISA Kit and Rat Tumour Necrosis Factor-α (TNF-α) ELISA Kit were used to quantify the IL-6 and TNF-α

### 2.8. Apoptosis Assay

Annexin V-FITC Apoptosis Detection Kit was used to detect apoptotic cells. Then, 24 h pre-treated cells were probed with annexin V and propidium iodide, as per the manufacturer’s protocol. Detection and quantification of apoptotic cell populations were performed using the BD FACSCalibur flow cytometry system (BD Biosciences, Franklin Lakes, NJ, USA). Each sample was analysed by measuring at least 20,000 cells within the gated region, with all experiments conducted in triplicate.

### 2.9. ROS Detection

The DCFH-DA-based redox probe was used to assess intracellular ROS activity through a fluorogenic assay. Briefly, using serum-free medium, 24 h pre-treated cells were washed and incubated with 1 μM DCF-DA for 60 min at 37 °C. The DCF-DA-containing medium was subsequently replaced with new fresh medium. DCFDA-probe cells were visualised and imaged with a Nikon ECLIPSE Ti-S inverted microscope using the green fluorescence channel. Plate reader-based fluorometric assay quantification using λ_ex_ 504 nm and λ_em_ 524 nm was performed to measure the DCF-DA intensity of the probed cells. Data collection and statistical calculation were carried out using three independent experimental replicates.

### 2.10. Nucleus Staining with Hoechst 33342

The nuclei were stained with Hoechst 33342 to assess apoptosis by observing nuclear morphology. Pre-treated cells were probed using 1 μM Hoechst 33342 for 10 min at 37 °C in dark. Finally, stained nuclei were observed using the Zeiss Axio Observer. Z1 inverted microscope system. The images were captured from three independent experiments.

### 2.11. Mitochondrial Membrane Potential (MMP) Detection

Rhodamine 123 (Rh123) probe was used to assess the electrical potential across the inner mitochondrial membrane. H9c2 cells pre-treated for 24 h were washed with PBS and probed with 10 µg/mL Rh123 in 2% FBS containing fresh media for 60 min at 37 °C in the dark. Subsequently, the visualisation and imaging of the washed cells were undertaken using a Nikon ECLIPSE Ti-S inverted microscope with a green fluorescence channel (Tokyo, Japan). Rh123 intake was quantified using plate reader-based fluorometric assay at λ_ex_ 488 nm and λ_em_ 530 nm. Data were collected from experimental triplicates.

### 2.12. Intracellular Calcium Measurement

Intracellular calcium (Ca^2+^) levels were measured utilising the ratiometric, Ca^2+^-sensitive dye, Fluo-4-AM. For this, 24 h pre-treated H9c2 cells were washed and probed with 5μM Fluo-4-AM in fresh culture media with 2% FBS at 37 °C for 30 min in a dark environment. Thereafter, cells were washed with PBS thrice and observed by Zeiss Axio Observer Z1 fluorescence microscope using the green channel. Fluorescent intensity of the Fluo-4-probed H9c2 cells was quantified using plate reader-based fluorometric assay at λ_ex_ 485 nm and λ_em_ 520 nm. Data were collected from experimental triplicates.

### 2.13. Ethics Declarations

All zebrafish-based experiments were conducted in accordance with the guidelines approved by the National Institute of Health Guidelines for the Institutional Animal Care and Use Committee of Nanyang Technological University, Singapore. For the experiment with larvae up to 120 hpf, the agreement of the Local Ethical Commission is not required.

All mice experiment protocols and procedures were approved by the Animal Care and Welfare Committee of the Institute of Materia Medica, Chinese Academy of Medical Sciences, and Peking Union Medical College, Beijing, China (Animal Experimental Centre, Institute of Materia Medica, CAMS and PUMC, Animal Experimental Ethical Inspection Approval No. 00006235).

### 2.14. Zebrafish Maintenance and Chemical Treatment

Wild-type zebrafish and transgenic zebrafish line Tg(cmlc2:gCaMP) [29] expressing a calcium-sensitive green fluorescent protein (GFP) in cardiomyocytes were utilised to visualise and evaluate the cardiac functions of zebrafish embryos in this study. Zebrafish were maintained in standard laboratory environments at 28 °C. Zebrafish embryos were collected following natural mating. The normal embryos without congenital morphological defects were selected after 24 hpf and 15 embryos were transfered into each well of the 24-well plate. Embryos were treated with Sham control and DOX with or without TP1. The treatment media were refreshed every 24 h. Fish larvae were subjected to treatment for 24 h (48 hpf), 48 h (72 hpf), and 72 h (96 hpf). Immediately after subsequent treatment, larvae were euthanised and examined, and single-plane wide-field epifluorescence images were taken by fluorescent microscope. Experiments were performed in three cohorts. A 0–100 µM DOX dose range was studied, and 50 μM DOX was used for final studies.

### 2.15. Morphological Abnormalities Assessment

Morphological assessments were performed throughout the experiment by examining the larvae under a light microscope. Cardiac abnormalities were examined for cardiotoxicity, including pericardial oedema and stretched heart. Severe malformations, including head and tail malformation and stunted growth, were also studied for toxicity. The fish larvae were immobilised with 3% methylcellulose and imaged using a fluorescence microscope (Axiovert 35, Zeiss, Oberkochen, Germany).

### 2.16. Cardiac Functionality Assessment

Six embryonic fishes per treatment were randomly selected for heart rate measurement at 48 hpf, 72 hpf, and 96 hpf. The larvae were allowed to acclimate at room temperature for 30 min, after which their heartbeats were observed and documented over a 20 s period. The ImageJ plugin Time Series Analyser V3 was used to analyse the Heartbeat videos.

Additionally, for evaluation of ventricular functions, transgenic zebrafish larvae expressing GFP in heart muscle were examined using a fluorescence microscope (Axiovert 35, Zeiss). Additioanlly, 96 hpf pre-treated larvae were acclimatised, immobilised, and examined. A complete heartbeat, including diastole, systole, and intervening pause, was imaged and recorded for further analysis. The diastolic and systolic status of the ventricle were determined using the elevation of the mean GFP intensity. We use the variation of ventricle volume during diastole and systole to quantify the ejection fraction (EF), a representation of cardiac function. The EF was calculated as the percentage of the total amount of blood pumped out with each heartbeat utilising a frequently applied equation: (EF (%) = (V_diastole_ − V_systole_)/(V_diastole_) × 100), where V_diastole_ represents diastolic ventricular diameter and V_systole_ refers to systolic ventricular diameter. The parameters were determined as the average of three separate measurements taken for each fish.

### 2.17. Doxorubicin-Induced Acute Myocardial Injury in Mice and Cardiac Functions Assessment

Clean-grade male ICR mice of 18–20 g (resident body weight) were acquired from the Weitong Lihua Experimental Animal Technology Centre, Beijing, China, and acclimatised for 2 days. On experiment Day 1, the mice were randomly assigned into groups consisting of 8 mice each, anaesthetised with isoflurane gas, and connected to a Biopac multi-channel physiological recorder (ECG100C electrocardiograph amplifier, BIOPAC Systems, Goleta, CA, USA) for monitoring and recording of electrocardiograms (ECG) and body weight through limb lead II. Mice in each group were then orally administered with resveratrol (RES) (50 mg/kg) or intraperitoneally (IP) injected with ginsentide TP1 (20 mg/kg), while the Sham control and doxorubicin (DOX) model group received equivalent volumes of pure water orally or normal saline via intraperitoneal injection, respectively. The treatment was given once daily for 5 consecutive days. On experiment Day 2, the model, RES, and TP1 group mice were injected 15 mg/kg DOX at a volume of 0.1 mL/10 g body weight intraperitoneally one hour after RES or TP1 administration, respectively. The body weight of the animals was regularly tracked during the experiment. The ECGs were performed and recorded on Day 1, 3, and 5 of the experiment. On experiment Day 5, following ECG recording, blood samples were collected for serum preparation. Serum concentrations of creatine kinase isoenzyme (CK-MB) and lactate dehydrogenase (LDH) were estimated using an automated biochemical analyser (MINDRAY BS-240, Mindray, Shenzhen, Guangdong, China). Collected data were processed, and the mean and standard error of each group were calculated. A *t*-test was adopted to compare the model group with the other groups. *p* < 0.05 was considered statistically significant.

#### Grouping

Control group: mice (n = 8) receiving Sham treatment for 5 days.

Model group: mice (n = 8) receiving DOX (15 mg/kg/day, i.p. at a volume of 0.1 mL/10 g body weight for 4 days).

RES 50 mg/kg group: mice (n = 8) receiving RES (50 mg/kg/day, oral for 5 days) and from Day 2, received DOX (5 mg/kg/day, i.p. for 4 days).

TP1 20 mg/kg group: mice (n = 8) receiving TP1 (20 mg/kg/day, i.p. for 5 days) and from Day 2, received DOX (5 mg/kg/day, i.p. for 4 days).

### 2.18. Statistical Analyses

The statistical analysis of data derived from triplicate experiments, or as specified, was conducted using GraphPad Version 6.01 software. Analysis of variance (ANOVA) with Šidák’s multiple comparisons test or Tukey’s multiple comparisons test method was used for statistical analysis and maintained in the respective figures. Data are presented as the mean ± standard deviation (SD) or standard error (SE) or standard error of the mean (SEM) as maintained. The threshold for statistical significance was set at *p* < 0.05.

## 3. Results

### 3.1. TP1 Prevents DOX-Induced Cytotoxicity and Increases the Survival of Cardiomyocytes

Ginsentide TP1 was extracted from dried flowers of *Panax ginseng* using water. The supernatant after clarification by centrifugation and membrane filtration was purified through ion exchange and multiple rounds of C18 reversed-phase high-performance liquid chromatography (RP-HPLC), as reported previously by our laboratory [30,31,32]. Its identity was confirmed unambiguously by mass spectrometry (Figure S1A,B). Purified ginsentide TP1 was used in all experiments performed in this report.

**Figure 2:**
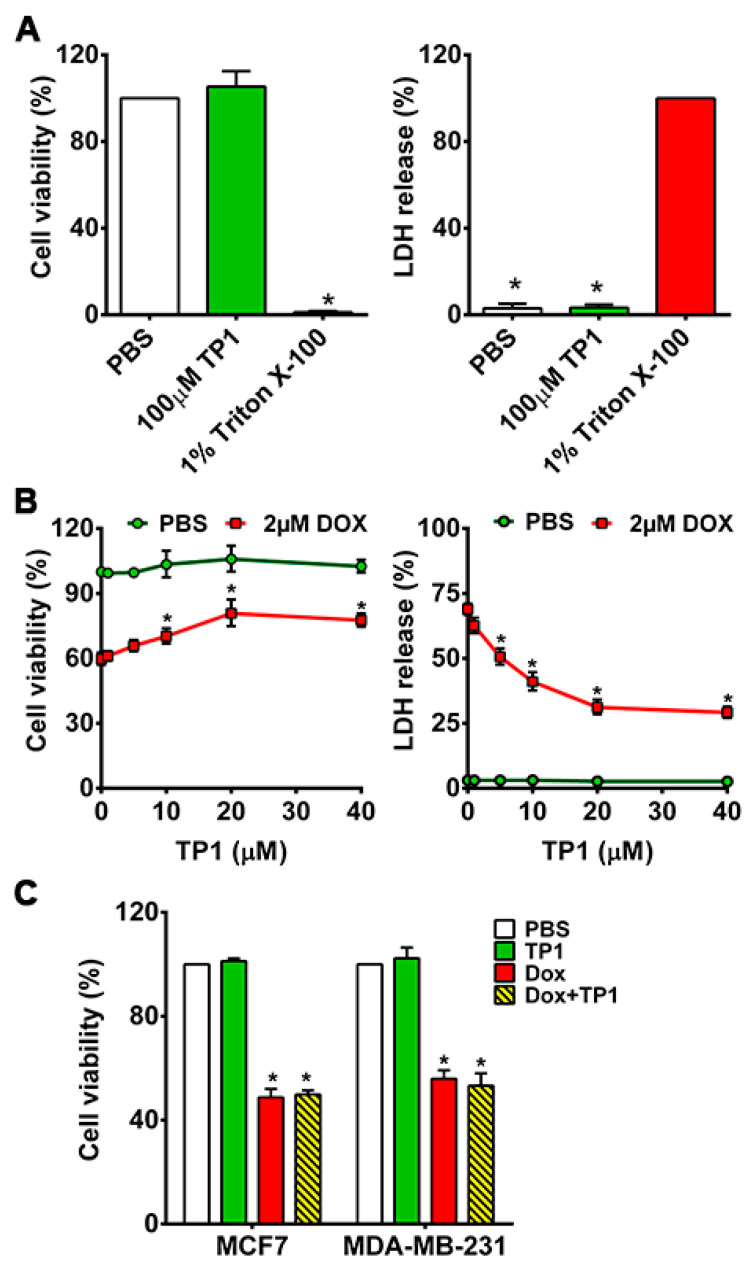
TP1 is nontoxic and prevents DOX-mediated cytotoxicity in H9c2 cardiomyocytes. (**A**) TP1 is nontoxic and nonmembranolytic—MTT-based cell viability analysis of H9c2 cells treated with 100μM of TP1 for 24 h. PBS was used as vehicle control, and 1% Triton X-100 (TX-100) was used as a positive control for cell death. n = 3; * *p* < 0.05 compared to PBS (left panel). LDH release-based cytotoxicity and membranolytic analysis of cells treated with 100 μM of TP1 for 24 h. n = 3; * *p* < 0.05 compared to 1% TX-100 (right panel). (**B**) TP1 prevents DOX-mediated cell death and increases cell viability. Cells were treated with PBS or 2 μM DOX with or without TP1 for 24 h, and viability was measured using an MTT assay. Data were presented as the percentage viability of the PBS control group. Data were presented as mean ± SEM (n = 4, analysis of variance (ANOVA) with Sidak’s multiple comparisons test). * *p* < 0.05 vs. only the DOX-treated group (left panel). TP1 mitigates DOX-induced cytotoxicity and membranolysis. A post-treated cell culture medium was assayed for LDH release. Data were represented as a percentage of LDH release in 1% Triton X 100-treated cells. Data were presented as mean ± SEM (n = 4, ANOVA with Sidak’s multiple comparisons test). * *p* < 0.05 vs. only the DOX-treated group (right panel). (**C**) Ginsentide TP1 has no impact on the anticancer effect of DOX in breast cancer cells. MDA-MB-231 and MCF7 cells were treated with PBS or 2 µM DOX with or without 10 µM TP1 for 24 h. Post-treated cells were subjected to an assessment of cell viability using an MTT assay. Cell viability is expressed as a percentage of PBS-treated cells. Data were represented as the mean ± SEM (n = 5, ANOVA with Tukey’s multiple comparisons test). * *p* < 0.05 vs. PBS control cells.

Previously, we showed that ginsentides and TP1 are nontoxic to different cell types [30,31,32]. To show that TP1 is nontoxic to cardiomyocytes, we incubated H9c2 cells with 100 µM TP1 for 24 h. No significant cytotoxicity was found using MTT and LDH release assays (Figure 2A), which supports our earlier findings that TP1 is nontoxic at concentrations ≤ 100 µM.

To study the in vitro effects of TP1, we next examined TP1-treated H9c2 cells with and without DOX for 24 h. We measured the IC_50_ of DOX (Figure S2A) and used 2 µM DOX for all assays in this study. The co-administration of TP1 and DOX results in a dose-dependent reduction in the DOX-induced cytotoxicity in H9c2 cells (Figure S2B and Figure 2B). The EC_50_ of TP1, evaluated in DOX-treated H9c2 cells (Figure S2B), demonstrated a significant enhancement in cell viability, reaching 72% upon 24 h co-treatment with 10 µM TP1 (Figure 2B and Figure S3). Hence, 10 µM TP1 was used as a representative dosage in all subsequent experiments.

Apart from MTT and LDH release assays, we noted pronounced alternation in the morphology of H9c2 cells. Following exposure to DOX, the cells became rounded and adhesion to cultured surfaces was reduced. Such morphological changes can be found in cells induced by genotoxic agents [38] (Figure S4A). No noticeable morphological changes were observed in TP1-treated cells, indicating that TP1 co-administration prevents DOX-induced morphological changes and preserves the regular cell morphology (Figure S4A).

### 3.2. TP1 Co-Treatment Does Not Compromise DOX Anticancer Efficacy

To investigate whether TP1 compromises the anticancer efficacy of DOX, we performed an MTT-based cell viability study using breast cancer cell lines MDA-MB-231 and MCF7. The viability of cancer cells was markedly reduced upon DOX treatment compared to PBS-treated cells (Figure 2C). Cancer cells co-treated with TP1 and DOX displayed similar cell viability as the DOX-treated groups, indicating that TP1 does not affect the anticancer effects of DOX (Figure 2C).

### 3.3. TP1 Inhibits DOX-Induced Apoptosis of H9c2 Cells

**Figure 3:**
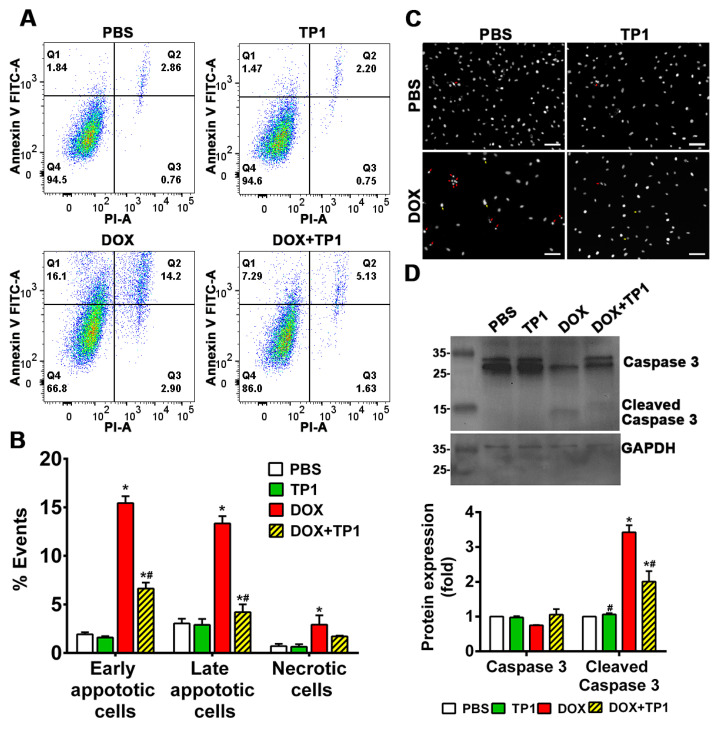
Anti-apoptotic effect of TP1 in DOX-treated H9c2 cardiomyocytes. Cells were treated with DOX for 24 h with or without TP1. Apoptotic cells in the post-treated samples were examined using annexin V/PI staining-based flow cytometric analysis. (**A**) Respective images show only annexin V-positive early apoptotic cells (Q1), both annexin V and PI-positive late apoptotic cells (Q2), only PI-positive necrotic cells (Q3), and normal (Q4) cell population. (**B**) Apoptotic and necrotic cell populations in each group were expressed as a percentage of the total 20,000 cell population. Data presented as mean ± SEM (n = 3, analysis of variance (ANOVA) with Sidak’s multiple comparisons test). * *p* < 0.05 vs. PBS control group and ^#^ *p* < 0.05 vs. only DOX-treated group. (**C**) Microscopic images showing the nuclei morphology of H9c2 cells. Post-treated cells were stained with Hoechst 33342 and observed using a fluorescent microscope. DOX-treated cells displayed morphological changes such as fragmented nuclei (red arrow) and other distorted nuclei of varying nuclear shapes and sizes (yellow arrows) compared to the PBS/TP1-treated groups. Scale bars = 100 µm. (**D**) Representative Western blot images show that TP1 prevents caspase-3 activation in DOX-treated cells. Total and cleaved caspase-3 expression was observed using Western blot analysis. Data in each group were normalised and expressed as a fold change in protein expression of the PBS-treated control group. Data presented as mean ± SEM (n = 3, ANOVA with Sidak’s multiple comparisons test). * *p* < 0.05 vs. PBS control group and ^#^ *p* < 0.05 vs. only DOX-treated group.

DOX-induced cell death is directly linked with apoptosis. The annexin V/PI staining technique was adopted to evaluate the anti-apoptotic effect of TP1 in the DOX-treated group. A flow cytometric analysis of annexin V/PI-stained cells revealed that DOX treatment significantly increases early apoptotic cell (Q1), late apoptotic cell (Q2), and necrotic cell (Q3) populations with respect to the untreated group (Figure 3A,B). The co-treatment of TP1 with DOX significantly reduced the numbers of apoptotic and necrotic H9c2 cells (Figure 3A,B). Similar to the LDH release assays described in the previous section, co-treatment with TP1 and DOX reduced the number of PI-stained cells (Figure S4B), indicating the preservation of the membrane integrity and anti-apoptotic effect.

To support the annexin V/PI staining results, we performed Hoechst 33342 staining. Our findings revealed that DOX-treated cells exhibited apoptotic features, including chromatin condensation and nuclear fragmentation. In contrast, the TP1-treated group predominantly displayed cells with minimal nuclear abnormalities (Figure 3C).

Cleaved caspase-3 is a protein biomarker for apoptosis. We examined DOX-induced apoptosis-cleaved caspase-3 levels in H9c2 cells by Western blot. The blotting results revealed that cleaved caspase-3 expression was significantly increased in DOX-treated cells (Figure 3D). In contrast, the expression of cleaved caspase-3 was significantly decreased in cells co-treated with TP1 and DOX (Figure 3D). Together, these results showed that TP1 mitigates DOX-mediated apoptosis in H9c2 cells to prevent DOX-induced cardiotoxicity.

### 3.4. TP1 Modulates Intracellular Calcium (Ca^2+^) Homeostasis in DOX-Treated H9c2 Cells

DOX-induced cardiotoxicity causes calcium dysfunction [36,39]. DOX disrupts calcium homeostasis by modulating the Ca^2+^ATPase and sodium/potassium exchanger activity in the sarcoplasmic reticulum and sarcolemma, respectively [39]. This DOX-dependent change in activity increases intracellular calcium loads in cardiac cells, enhancing the generation of ROS. Ultimately, this leads to an increase in oxidative stress and cellular damage, resulting in cardiotoxicity [40].

**Figure 4:**
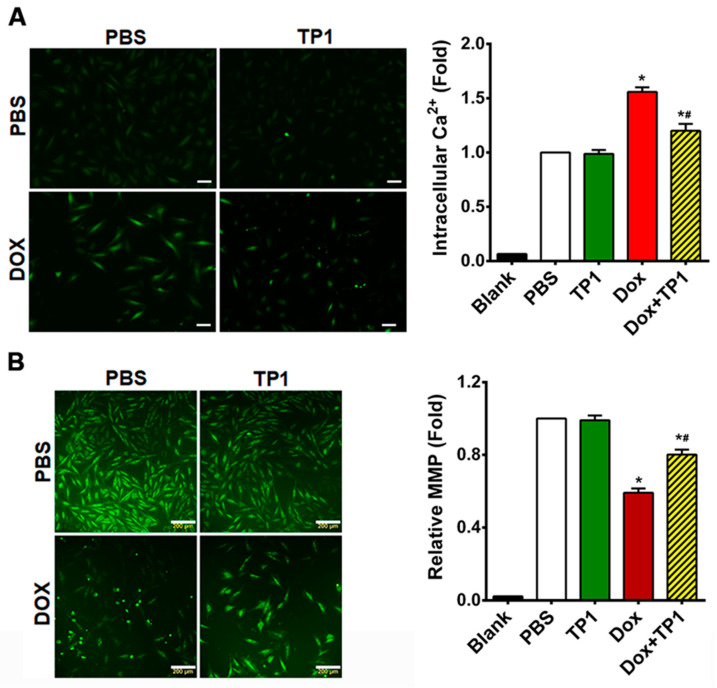
TP1 restores intracellular calcium (Ca^2+^) homeostasis and augments mitochondrial membrane potential (MMP) in DOX-treated H9c2 cells. (**A**) Representative microscopic images show that TP1 co-administration reduces DOX-mediated Ca^2+^ overload. Intracellular Ca^2+^ content was observed and calculated using a ratiometric Fluo-4-AM probe. Intracellular Ca^2+^ level was presented as the relative intensity of the Fluo-4 signal to PBS-treated control cells. Data were presented as representative microscopic images and mean values (±SEM) of about 100 cells in each group from experimental triplicates (analysis of variance (ANOVA) with Tukey’s multiple comparisons test). * *p* < 0.05 vs. PBS control group and ^#^ *p* < 0.05 vs. only DOX-treated group. Scale bars, 50 µm. (**B**) Representative microscopic images show that TP1 co-treatment restores MMP in DOX-treated cells. MMP of H9c2 cells was measured and quantified by Rhodamine 123 uptake. Scale bars = 200 µm. The relative MMP of respective experimental groups was calculated using ratiometric Rhodamine 123 uptake. Data in each group were normalised and expressed as a fold of the PBS-treated control group. Data were shown as mean values (± SEM) (n = 3, ANOVA with Tukey’s multiple comparisons test). * *p* < 0.05 vs. PBS control group and ^#^ *p* < 0.05 vs. only DOX-treated group.

To study how TP1 treatment affects intracellular calcium homeostasis, we assessed the intracellular Ca^2+^ content with a Fluo-4-AM probe. Microscopic images and the fluorometric quantification of intracellular Ca^2+^ concentrations in H9c2 cells showed that DOX-treated cells increased 1.5-fold in fluorescent intensity compared to PBS-treated H9c2 cells (Figure 4A), suggesting that DOX significantly elevates the intercellular Ca^2+^ load. In contrast, cells treated with TP1 alone showed a fluorescent profile similar to that of PBS-treated control cells (Figure 4A). However, the co-administration of TP1 and DOX significantly reduced the intracellular Ca^2+^ concentration, as shown by a lower fluorescent intensity compared to DOX-treated cells (Figure 4A). Together, these results support TP1 playing an important role to modulate intracellular homeostasis in DOX-treated and mitochondria-rich cardiomyocytes.

### 3.5. TP1 Restores MMP in DOX-Treated H9c2 Cells

The binding of DOX with cardiolipin, an inner mitochondrial membrane phospholipid, results in mitochondrial dysfunction, which significantly contributes to DOX-induced cardiotoxicity [41]. Thus, we examined the effects of TP1 on the altered mitochondrial membrane potential (MMP) caused by DOX [41,42,43]. The altered MMP in H9c2 cells was assessed using Rhodamine 123, which is taken up by normal, active mitochondria to yield green fluorescence signals. In contrast to the untreated cells, DOX-treated cells exhibited a markedly weaker green fluorescence signal, indicating an altered MMP (Figure 4B). TP1 and DOX co-administration reversed this alternation pattern of fluorescence, suggesting that the MMP in cells was partially restored by TP1 (Figure 4B). Quantitative fluorescence intensity analysis from plate reader-based experiments revealed that these fluorescence alterations were significant (Figure 4B). Our results suggest that the mitigation of DOX-mediated mitochondrial dysfunction is an underlying mechanism by which TP1 prevents DOX-induced cardiotoxicity.

### 3.6. TP1 Reduces DOX-Mediated ROS Production in H9c2 Cells

**Figure 5:**
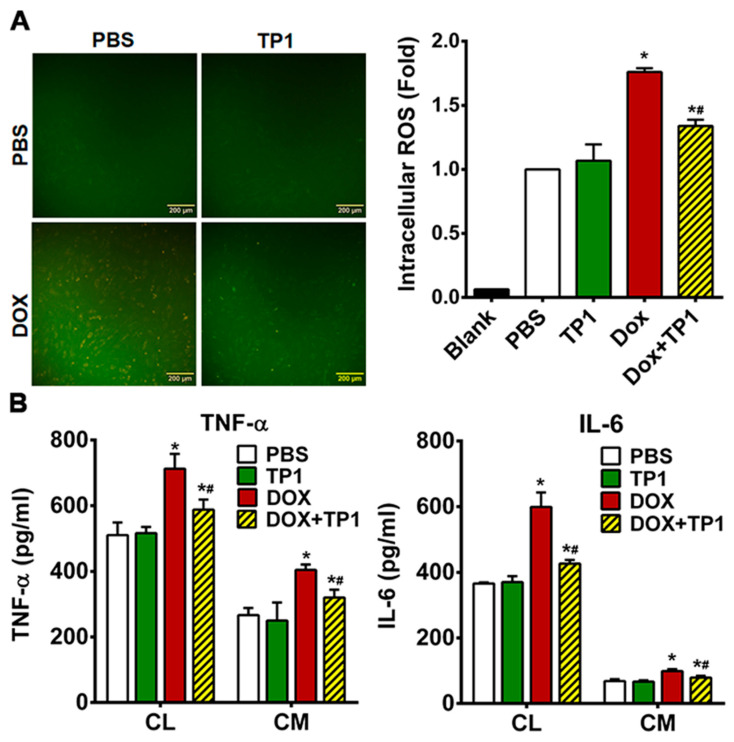
TP1 mitigates oxidative stress-linked inflammatory responses in DOX-treated H9c2 cells. (**A**) TP1 inhibits DOX-mediated ROS generation in H9c2 cells. Cells were treated with PBS or DOX with or without TP1 for 24 h, and ROS activity was observed using a DCFH-DA redox probe. Scale bar = 200 µm. Relative ROS levels were estimated through DCFH-DA-based fluorometric assay. Data were presented as mean ± SD (n = 3, analysis of variance (ANOVA) with Tukey’s multiple comparisons test). * *p* < 0.05 vs. PBS-treated control groups and ^#^ *p* < 0.05 vs. DOX-treated groups. (**B**) TP1 prevents DOX-induced inflammatory responses in H9c2 cells. Proinflammatory cytokines’ TNF-α and IL-6 expression and secretion were estimated with ELISA using respective cell lysates (CL) and condition media (CM). Data were presented as mean values (±SEM) (n = 3, ANOVA with Sidak’s multiple comparisons test). * *p* < 0.05 vs. PBS-treated control groups and ^#^ *p* < 0.05 vs. DOX-treated groups.

DOX-induced cardiotoxicity is primarily linked with ROS generation and oxidative stress-mediated cellular damage. To evaluate the influence of TP1 on the generation of ROS, we examined the intracellular ROS levels in H9c2 cells using the redox probe DCFH-DA. A yellowish-green fluorescence is visible in microscopic images of DCFH-DA-loaded H9c2 cells. Both the untreated control and TP1-treated cells exhibited very weak green fluorescence, while DOX-treated cells produced a strong yellowish-green fluorescence (Figure 5A). In contrast, the fluorescent signals in cells co-treated with TP1 and DOX were significantly reduced when compared to those cells treated with DOX alone (Figure 5A). A fluorometric quantification revealed that DOX-treated H9c2 cells showed a substantial rise in intercellular ROS levels (1.8-fold) compared to untreated H9c2 cells (Figure 5A). In comparison, DCFH fluorescence decreased 1.4-fold in TP1, and DOX co-administered cells with respect to only DOX-treated cells (Figure 5A). This result shows a substantial drop in DOX-mediated ROS generation in the presence of TP1. These observations suggest that TP1 co-administration prevents DOX-induced ROS production and oxidative stress, promoting the mitigation of DOX-induced cardiotoxicity.

### 3.7. TP1 Pre-Empts DOX-Induced Inflammatory Responses in H9c2 Cells

DOX-induced cardiotoxicity is linked with increased inflammatory responses [44,45,46]. To determine whether TP1 has an anti-inflammatory effect, we used ELISA to measure the production and release of proinflammatory markers in DOX-treated H9c2 cells. Our results showed an increased expression and secretion of tumour necrosis factor-α (TNF-α) and interleukin 6 (IL-6) in H9c2 cells treated with DOX (Figure 5B). In contrast, the co-administration of TP1 and DOX showed a reduced expression and secretion of proinflammatory cytokines including IL-6 and TNF-α in H9c2 cells (Figure 5B). These results suggest that TP1 can mitigate inflammatory responses induced by DOX.

### 3.8. TP1 Mitigates DOX-Induced Cardiotoxicity in Zebrafish

**Figure 6:**
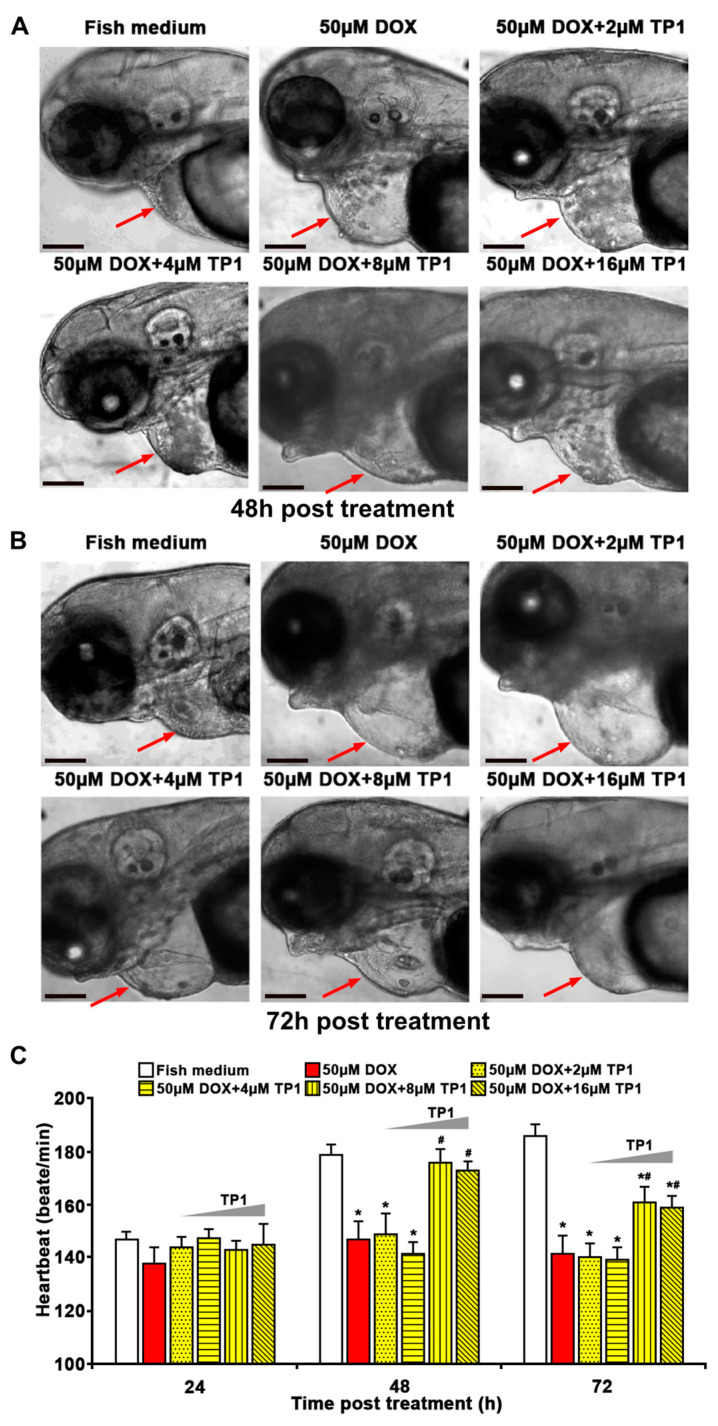
Ginsentide TP1 mitigates DOX-induced cardiotoxicity and restores cardiac function in zebrafish. In total, 24 hpf zebrafish embryos were used to visualise and evaluate the cardiac functions and 24 hpf embryos were treated with Sham control (Fish medium) or 50 µM of DOX with or without TP1 co-treatment. Six randomly selected larvae for each group from three cohorts were used for assessment. (**A**,**B**) Representative images of zebrafish embryos after 48 h and 72 h post-treatment. Red arrows indicate the cardiac sacs of zebrafish embryos. Enlarged cardiac sacs were detected in DOX-treated embryos, indicating the presence of pericardial oedema. Scale bar = 100 μm. (**C**) TP1 restores the heartbeat rates of DOX-treated zebrafish. The heartbeat of individual embryos was measured at 24 h, 48 h, and 72 h post-treatment. Data are presented as means ± SEM of 3 independent experiments (n = 45, analysis of variance (ANOVA) with Sidak’s multiple comparisons test). * *p* < 0.05 vs. Sham control group and **^#^** *p* < 0.05 vs. DOX-treated group.

To support our in vitro experiments, we investigated the cardioprotective function of TP1 in both wild-type and transgenic Tg(cmlc2:gCaMP) [29] zebrafish. The transgenic zebrafish expresses a calcium-sensitive green fluorescent protein (GFP) in cardiomyocytes, enabling the assessment and visualisation of the cardiac function. The zebrafish group treated with DOX showed severe cardiomyopathy, including reduced heart rates, ventricular contractility, and pericardial oedemas with inflamed heart sacs (Figure 6A–C and Figure S5A,B and Videos S1–S4). In contrast, zebrafish embryos treated with escalating TP1 concentrations up to 32 µM showed no observable abnormality (Figure S5C,D). The DOX toxicity was dose-dependent, and the fish died 72 h after treatment with 100 µM DOX (Figure S5A,B and Videos S1–S4). However, the co-administration of TP1 and DOX prevented the detrimental effects of DOX and restored the heart rate to near normal (Figure 6A–C and Videos S1–S4). Moreover, TP1 and DOX co-treatment significantly reduced DOX-induced pericardial oedemas in embryos, which was shown by the reduced size of the cardiac sacs compared to the highly enlarged cardiac sacs seen in the fishes treated with DOX (Figure 6A,B).

**Figure 7:**
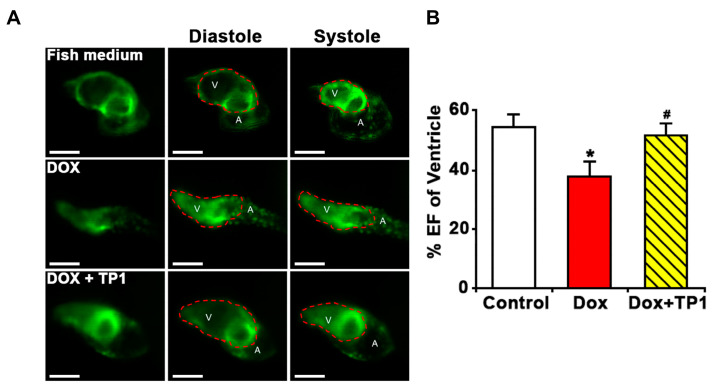
Ginsentide TP1-cotreatment prevents DOX-induced ventricular dysfunction in zebrafish. Zebrafish transgenic line Tg (cmlc2: gCaMP) expressing a calcium-sensitive green fluorescent protein (GFP) in cardiomyocytes was used to visualise and evaluate the cardiac functions of zebrafish embryos. Twenty-four hpf embryos were treated with fish medium (Sham control) or 50 µM of DOX with or without TP1 co-treatment for 72 h. Data were collected from three cohorts, and six embryos were selected randomly from each group. (**A**) Representative images of hearts of pre-treated zebrafish embryos. Images show ventricular contractility of 72 h post-treated embryos. ‘V’ represents the ventricle and ‘A’ represents the atrium. Red dotted lines indicate the outlines of the ventricles. Scale bar = 50 μm (**B**) The ventricular ejection fraction (EF) was calculated from diastolic and systolic volume after 72 h post-treatment. Data were presented as means ± SEM (n = 18, analysis of variance (ANOVA) with Sidak’s multiple comparisons test). * *p* < 0.05 vs. Sham control group and # *p* < 0.05 vs. DOX-treated group.

Additionally, the ventricular functions of zebrafish embryos were evaluated based on the ejection fraction (EF). Likewise, zebrafish in the TP1 and DOX co-administered group improved the DOX-induced reduction of EF (Figure 7A,B and Videos S5–S7). Thus, the in vivo results in the zebrafish model agree with our in vitro results, showing that TP1 can confer significant protection against cardiotoxicity induced by DOX.

### 3.9. Cardioprotective Property of TP1 in Mitigating DOX-Induced Cardiotoxicity in a Murine Model

**Figure 8:**
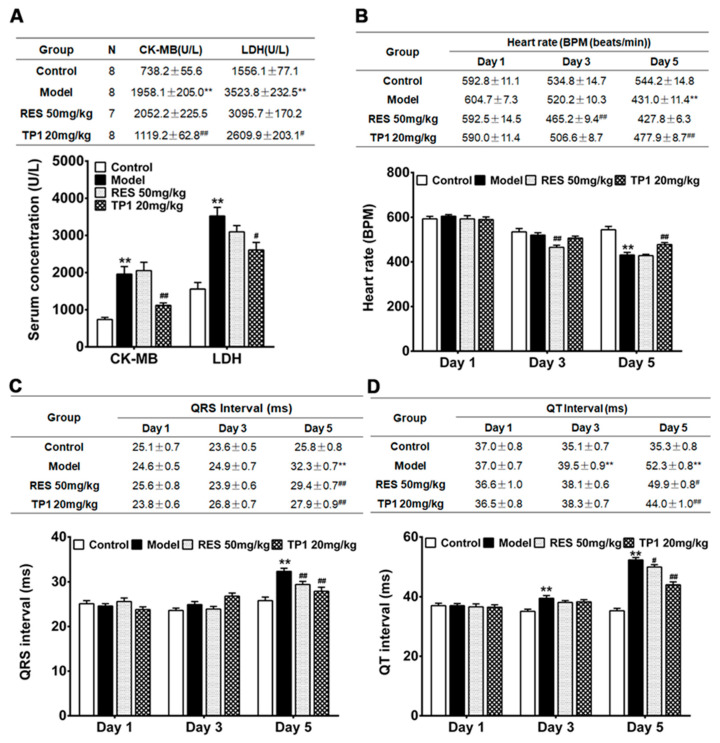
Intraperitoneal administration of ginsentide TP1 mitigates DOX-induced acute myocardial injury in mice. Male ICR mice were prophylactically treated with resveratrol (RES) (50 mg/kg) and TP1 (20 mg/kg), one dose per day for 5 days. On experiment Day 2, 1 h after administration of RES or TP1, DOX was injected intraperitoneally to induce acute myocardial injury. On experiment Day 1, 3, and 5, the ECG of the animals were recorded, and serum was withdrawn accordingly. Eight mice were used for each group. Data represented as mean and standard error (±SE) and significance were calculated using analysis of variance (ANOVA) with Sidak’s multiple comparisons tests. (**A**) Ginsentide TP1 reduces serum levels of creatine kinase MB isoenzyme (CK-MB) and lactate dehydrogenase (LDH) in DOX-induced acute myocardial injury in mice (** *p* < 0.01 vs. control, ^#^ *p* < 0.05 and ^##^ *p* < 0.01 vs. model). (**B**) Oral or intraperitoneal administration of RES and TP1, respectively, improved the heart rate of DOX-induced acute myocardial injury mice (** *p* < 0.01 vs. control, ^##^ *p* < 0.01 vs. model). (**C**) Oral or intraperitoneal administration of RES and TP1, respectively, restored the QRS interval in DOX-treated mice (** *p* < 0.01 vs. control, ^##^ *p* < 0.01 vs. model). (**D**) Oral or intraperitoneal administration of RES and TP1, respectively, shortened the QT interval of DOX-treated myocardial-injured mice (** *p* < 0.01 vs. control, ^#^ *p* < 0.05, ^##^ *p* < 0.01 vs. model).

We further evaluated the in vivo cardioprotective property of TP1 in a murine model. An acute myocardial injury was induced in male ICR mice using DOX. Resveratrol, a phenolic phytoalexin, was used as a prophylactic reference compound. On experiment Day 1, resveratrol (50 mg/kg) was administered orally as a prophylactic, while TP1 (20 mg/kg) or PBS (Sham control) was administered intraperitoneally once daily for a duration of 5 days. However, PBS was administered to the only DOX-treated group (Model group) on Day 1. On Day 2, DOX was injected intraperitoneally to induce an acute myocardial injury. Compared with the Sham control animals, both serum LDH and CK-MB were substantially increased (*p* < 0.05) in the model group treated with DOX (Figure 8A), indicating a significant myocardial injury. In contrast, the prophylactic administration of TP1 serum levels of LDH and CK-MB were markedly reduced (*p* < 0.05) (Figure 8A), suggesting that TP1 prevents DOX-mediated myocardial injury.

Cardiac functions were evaluated by recording electrocardiograms (ECG) of the mice on experiments Day 1, 3, and 5, and their heart rate, QRS, and Q-T interval were determined accordingly. On Day 5, the ECG recordings in the DOX-treated group exhibited an acute myocardial injury, with significantly reduced (*p* < 0.05) heart rates and prolonged QRS and QT intervals compared to the Sham control mice (Figure 8B–D). Conversely, the TP1-treated group showed significant improvement in their heart rate, QRS, and QT intervals on the ECG recordings (*p* < 0.05) when compared to the model group treated with DOX (Figure 8B–D), suggesting that TP1 improves cardiac functions by protecting against DOX-induced acute myocardial injury.

## 4. Discussion

This work reports an important application of ginsentide TP1, a naturally occurring microprotein, to counter serious side-effects of cancer chemotherapy. We show that the recently discovered ginsentide TP1 protects against DOX-induced cardiotoxicity without impairing its anticancer efficacy. TP1, a member of the cysteine-rich superfamily, displays a highly compact structure that is distinctly different from the commonly found structural–flexible peptides of a similar size. The tightly disulphide-crossbraced microprotein structure of TP1 renders it resistant to proteolytic degradation and enables it to be orally bioavailable [31,32]. Importantly, TP1 is cell-permeable, which allows it to target intracellular proteins.

Previously, we showed that ginsentides have a broad-spectrum activity to relieve stress caused by external or internal stressors [31,32]. In this regard, ginsentides act as the principal adaptogenic and anti-stress compounds to produce the “cure-all” medicinal effects of *Panax ginseng*. To the best of our knowledge, this is the first report of ginsentide TP1 as a cardioprotective peptide that offers the potential to protect cancer patients from anthracycline-based chemotherapy.

The literature reports suggest that DOX-induced cardiotoxicity is influenced by multifaceted mechanisms such as ROS generation, oxidative stress, mitochondrial dysfunction, calcium dysregulation, inflammation, and apoptosis, all of which contribute to the progression of cardiomyopathy and heart failure [5,6,7,34,35,36,37,38,39,40,41,42,43,44,45,46,47,48,49,50,51]. In agreement with these reports, our in vitro study using H9c2 cardiomyocytes has confirmed that DOX indeed induces oxidative stress, elevating ROS levels, reducing MMP, increasing the expression and release of proinflammatory cytokines, including TNF-α and IL-6, and enhancing apoptotic activity leading to cell death. While antioxidants can mitigate DOX-induced oxidative stress-linked cardiotoxicity, effective therapeutic options are still limited due to the complexity of these conditions [17,21].

Previously, we reported that TP1 is a vasoprotective microprotein that prevents hypoxia-induced vascular endothelial dysfunction and apoptosis [30,31,32]. In line with this role, TP1 protects against DOX-induced cardiotoxicity. Co-treatment with DOX and TP1 in H9c2 cells significantly reduced ROS production, oxidative stress, and apoptosis, improved MMP, and inhibited the expression and release of proinflammatory cytokines including TNF-α and IL-6.

DOX-induced oxidative stress impairs the mitochondrial function and MMP mechanisms, disrupting the eNOS/NO pathway, affecting critical cellular functions, and triggering mitochondrial dysfunction, inflammation, and apoptosis [42,43,44,45,46,47,48,49,50,51,52,53,54]. Co-treatment with TP1 restores the eNOS/NO pathway activity and mitochondrial function [31,32], mitigating oxidative damage and reducing apoptosis in cells treated with DOX, as shown by the decreased population of apoptotic cells and reduced cleaved Caspase3 expression levels.

DOX also disrupts calcium homeostasis, affecting sarcoplasmic reticulum functions and promoting intracellular calcium release, which leads to ROS generation [36,37,38,39,40,44,55]. We also observed elevated ROS and intracellular calcium levels in DOX-treated cells, along with prior studies [43,44,45,46]. However, TP1 co-administration improved MMP and reduced ROS and calcium levels, suggesting that TP1 alleviates the harmful effects of DOX on the mitochondria. Our data showed DOX-induced dose-dependent cytotoxicity in H9c2 cells via apoptosis, a crucial factor in DOX-mediated cardiac dysfunction [42,43,44,45,46,47,48,49,50,51,52,53,54,55,56]. Thus, the anti-apoptotic property of TP1 prevents DOX-mediated cytotoxicity in H9c2 cells, as demonstrated by LDH release and MTT assays, without compromising the anticancer efficacy of DOX as a chemotherapeutic agent.

DOX can induce heart failure in animal models [56,57,58,59]. To confirm our in vitro results, we assessed the protective effect of TP1 in zebrafish and mouse models of DOX-induced cardiac injury. Consistent with earlier findings, DOX-treated zebrafish exhibited a decreased heart rate and ventricular ejection fraction, as well as induced pericardial oedemas, all of which are well-established clinical indicators of cardiotoxicity [57,58,59,60,61,62]. Importantly, TP1 co-treatment mitigated these DOX-induced detrimental effects, restoring normal cardiac function and protecting against cardiac injury.

**Figure 9:**
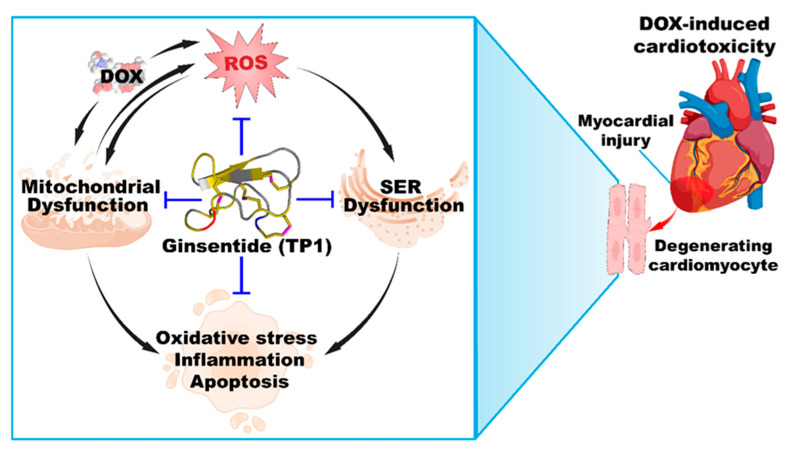
Ginsentide TP1 is cardioprotective and mitigates DOX-induced cardiotoxicity.

In DOX-induced cardiotoxicity, bradycardia, prolonged QRS, and broader QT intervals are often reflected in ECG records [53,63,64,65]. Our in vivo studies found a reduced heart rate and increased QRS and QT intervals in DOX-administered mice, leading to acute myocardial injury [53,66,67]. TP1 pretreatment counters these effects by improving the heart rate and shortening the QRS and QT intervals, confirming its cardioprotective potential. Moreover, elevated levels of serum CK-MB and LDH enzymes in DOX-treated mice strongly suggest myocardial injury, which TP1 mitigates [63,68,69]. These findings agree with previous studies showing that DOX-induced myocardial injury is greatly influenced by oxidative stress [36,37,38,39,40,41,42,43,44,45,46,47,70,71], which can be reversed by TP1 pretreatment. In summary, TP1 alleviates DOX-induced cardiotoxicity by targeting oxidative stress, an inflammatory response, and mitochondrial dysfunction in both in vivo and in vitro models (Figure 9). Together, these results indicate that TP1 holds potential as an effective pre-emptive treatment for mitigating DOX-induced cardiac damage.

Ginsentide TP1 differs from known cardioprotective amino acids and peptides such as acetylcysteine, reduced glutathione, and proteins such as erythropoietin (EPO) [41]. Both acetylcysteine and reduced glutathione are small thiol-containing compounds that act as antioxidants, while TP1 has no free thiol. In terms of size, TP1 is 10–30 times bigger than acetylcysteine or glutathione, but ten times smaller than EPO. Unlike EPO, which exerts its cardioprotective mechanism upon binding to its extracellular receptor [41], ginsentides act on extra- and intra-cellular targets. Previously, we reported that ginsentides induce vasorelaxation through the production of NO via the PI3K/Akt signalling pathway [31,32]. In addition, they mitigate α1-adrenergic receptor hyperactivity by counteracting phenylephrine-induced aortic constriction, leading to reduced monocyte attachment to endothelial cells through CD166/ESAM/CD40 and suppressing P2Y12 receptor activation to inhibit platelet aggregation [32]. We also demonstrated that TP1 safeguards endothelial cells from hypoxia-induced endothelial dysfunction, which is marked by ER stress, decreased NO bioavailability, and elevated ROS production [31].

In addition to the structural compactness, pseudocyclic arrangement (see the Figure 1 legend for explanation), and high stability against thermo-chemical and enzymatic degradation, TP1 can penetrate cells to interfere with intercellular protein–protein interactions [30,31,32]. Certain plant-derived cysteine-rich microproteins share this cell-penetrating property of TP1. They include members of the ginsentide family, 8C-hevein-like peptides, α-astratides, and β-ginkgotides [33,72,73]. Interestingly, we have recently isolated roseltide rT1, a mitochondria-targeting cysteine-rich peptide from *Hibiscus sabdariffa* that is also cell-penetrating and is transported into the mitochondria viaTOM20, a receptor involved in mitochondria protein imports [74]. Roseltide rT1 enhances ATP synthesis by promoting mitochondrial hyperpolarisation [74]. It would be interesting to see if TP1 could work with such an energy-boosting peptide to improve its adjuvant efficacy. TP1 is non-toxic and does not exhibit any cytotoxic, membranolytic, mitogenic, or mutagenic properties [30,31,32]. The exceptional physicochemical and metabolic stability, along with cell-penetrating activity [30,31,32], make ginsentides a promising family of stable microproteins for drug development.

## 5. Conclusions

In this study, we report that the ginsentide TP1 effectively mitigates DOX-induced cardiotoxicity by reducing oxidative stress and inflammation, thereby maintaining effective cardiac functions in chemotherapy. Notably, the adaptogenic and protective properties of TP at a lower dose (10μM) do not compromise the cytotoxic actions of DOX in breast cancer cells. We highlight the therapeutic potential of TP1 in addressing cardiotoxicity caused by DOX. Further studies are warranted to investigate its bioavailability, the dose–response relationships of TP1, the in vivo effect on DOX-induced myelotoxicity, and the tumour-suppressive efficacy of DOX for its use as a cardioprotectant in chemotherapy.

## Data Availability

This study does not involve an experimental dataset deposited in an external data depository.

## Supplementary Materials

The supporting information can be downloaded at: https://www.mdpi.com/article/10.3390/antiox14040493/s1.

## Abbreviations

The following abbreviations are used in this manuscript:

ACN	Acetonitrile
ANOVA	Analysis of variance
Ca^2+^	Calcium ion
CRPs	Cysteine-rich peptides
DOX	Doxorubicin
EF	Ejection fraction
ECG	Electrocardiograms
ER	Endoplasmic reticulum
eNOs	Endothelial nitric oxide synthase
ELISA	Enzyme-linked immunosorbent assay
EPO	Erythropoietin
GFP	Green fluorescent protein
IL-6	Interleukin 6
LDH	Lactate dehydrogenase
MMP	Mitochondrial membrane potential
NO	Nitric oxide
ROS	Reactive oxygen species
GSH	Reduced glutathione
RP-HPLC	Reversed-phase high-performance liquid chromatography
Rh123	Rhodamine 123
CK-MB	Serum concentration of creatine kinase isoenzyme
SER	Smooth endoplasmic reticulum
SD	Standard deviation
SE	Standard error
SEM	Standard error of the mean
TFA	Trifluoroacetic acid
TNF-α	Tumour necrosis factor-α
