# Supplementary material for: Cell-Permeable Microprotein from Panax Ginseng Protects Against Doxorubicin-Induced Oxidative Stress and Cardiotoxicity
Source: Antioxidants (Basel). 2025 Apr 19;14(4):493. doi: 10.3390/antiox14040493 (PMC12024455; doi:10.3390/antiox14040493)
Supplement: Supplementary file 1 [file antioxidants-14-00493-s001.zip › Supplementary Figures.pdf]

Cell-permeable microprotein from *Panax ginseng* protects against doxorubicin-induced oxidative stress and cardiotoxicity

Bamaprasad Dutta[1, 2], Shining Loo[1, 3], Antony Kam[1, 4], Xiaoliang Wang[1, 5], Na Wei[6], Kathy Qian Luo[7,8], Chuan-Fa Liu[1], and James P. Tam[1]*

[1]School of Biological Sciences, Nanyang Technological University, 60 Nanyang Drive, Singapore 637551, Singapore; bama0001@e.ntu.edu.sg (B.D.); shining.loo@xjtlu.edu.cn (S.L.); antony.kam@xjtlu.edu.cn (A.K.); wangxl@imm.ac.cn (X.W.); cfliu@ntu.edu.sg (C.-F.L.)
[2]School of Pharmacy, The Neotia University, Sarisa, Diamond Harbour Road, 24 Parganas (South), West Bengal 743368, India
[3]Wisdom Lake Academy of Pharmacy, Xi'an Jiaotong-Liverpool University, Suzhou 215123, China
[4]Department of Biological Sciences, Xi'an Jiaotong-Liverpool University, Suzhou 215123, China
[5]Institute of Materia Medica, Chinese Academy of Medical Sciences, Beijing 100050, China
[6]School of Chemistry, Chemical Engineering and Biotechnology, Nanyang Technological University, 70 Nanyang Drive, Singapore 637457, Singapore; na.wei@tu-darmstadt.de
[7]Faculty of Health Sciences, University of Macau, Taipa, Macao SAR, China; kluo@um.edu.mo
[8]Ministry of Education Frontiers Science Center for Precision Oncology, University of Macau, Taipa, Macao SAR, China
*Correspondence: jptam@ntu.edu.sg; Tel.: +65-88625722

Supplementary figures

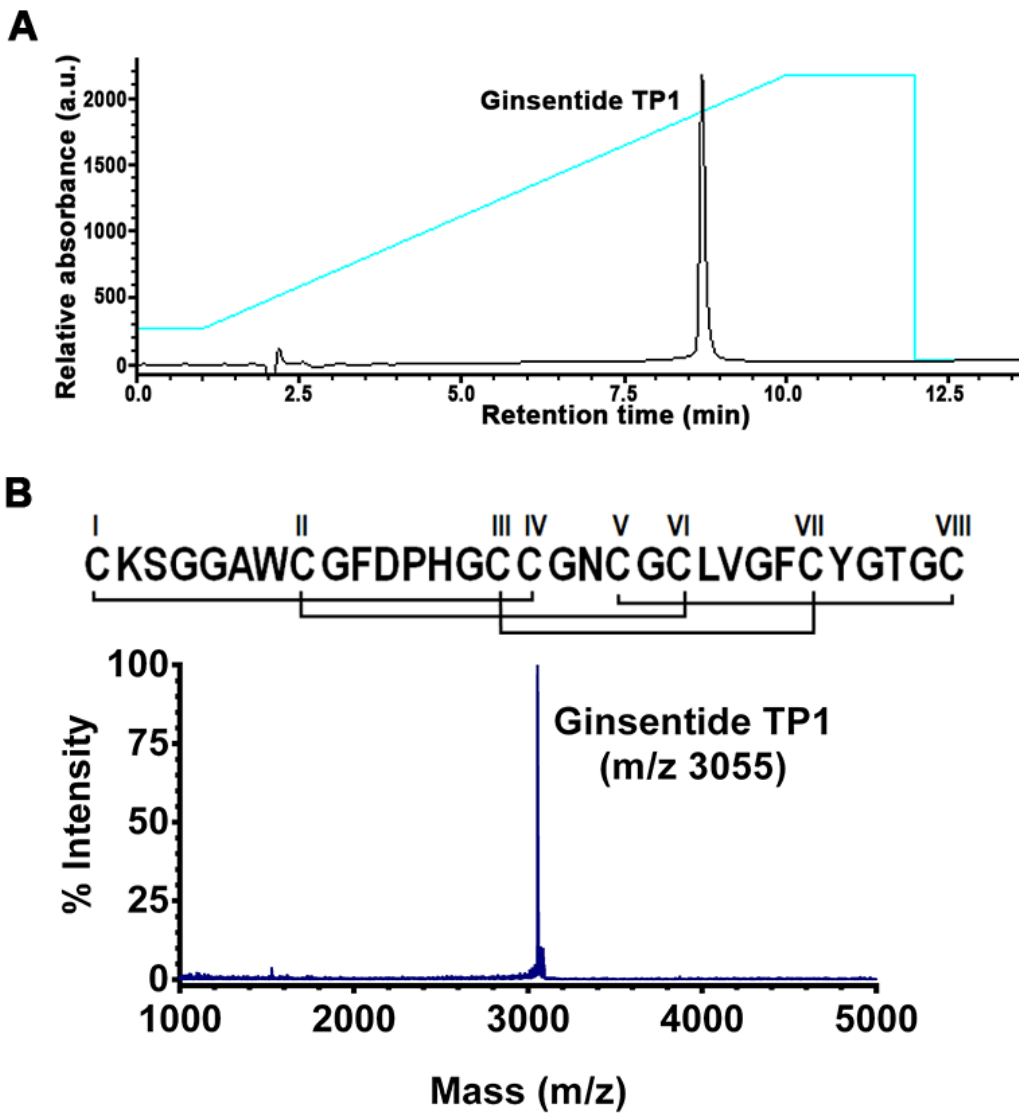

Figure S1: Analytical profiling of purified ginsentide TP1 extracted from *Panax ginseng* flowers. (A) HPLC chromatogram of purified TP1. (B) Mass spectrometry profile of purified TP1.

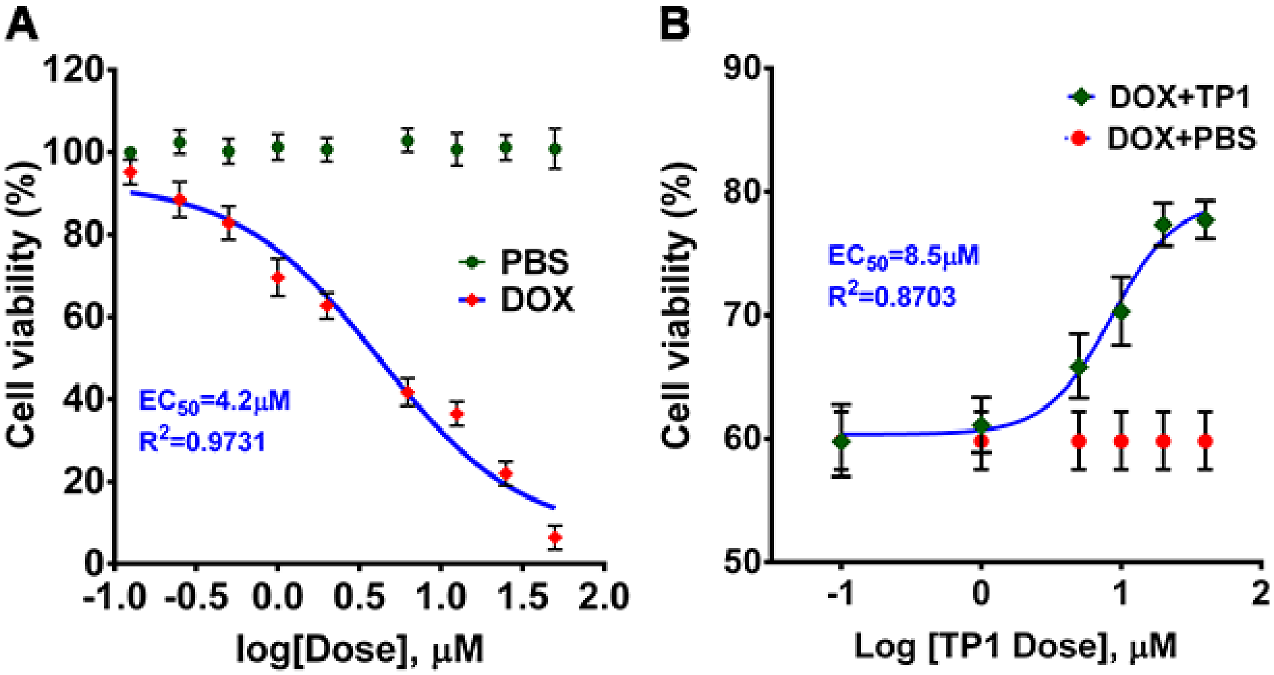

Figure S2: Dose-response of doxorubicin (DOX) and TP1 in H9c2 cells. (A) Cytotoxicity assessment of DOX in H9c2 cells. Cells were treated with DOX for 24h, and cell viability was assessed with an MTT assay. Data showed percentage viability compared to the PBS-treated group calculated from three independent experiments. (B) Dose-response of TP1 in DOX-mediated cell death. Cells were treated with mock (PBS) or DOX with or without TP1 for 24h, and cell viability was measured using an MTT assay. Data was presented as percentage viability of the PBS-only control group. Both data represent mean values with 95% confidence intervals (CI). The EC_{50} value was calculated using nonlinear regression with the best fit, and goodness of fit was used to calculate R^2.

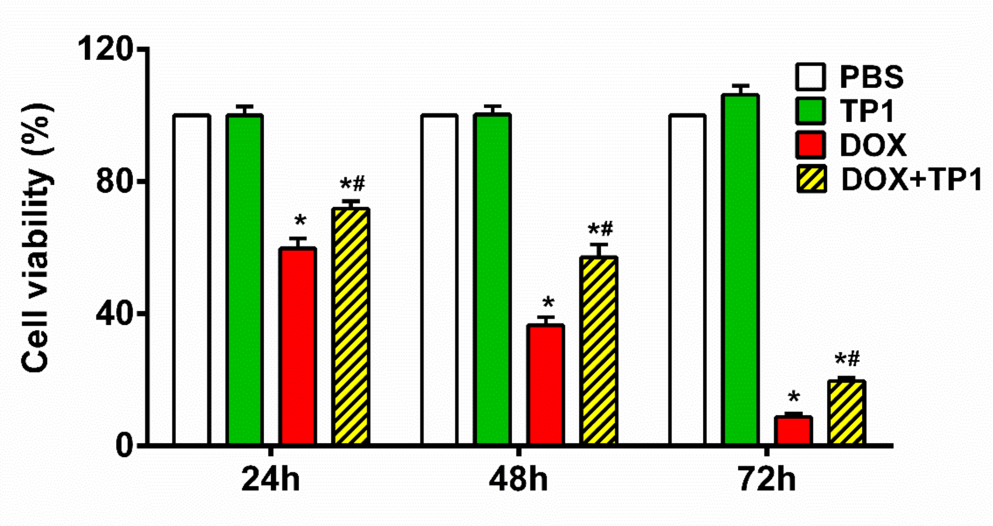

Figure S3: Ginsentide TP1 prevents DOX-mediated cytotoxicity in H9c2 cardiomyocytes. H9c2 cells were treated with PBS or 2μM DOX with or without TP1 for 24h, 48h, and 72h, respectively. Post-treated cells were subjected to an assessment of cell viability using an MTT assay, and data was represented as a percentage of PBS-only control groups. Data was presented as mean ± SEM (n=4, analysis of variance (ANOVA) with Tukey's multiple comparisons test). *p<0.05 vs. PBS control group and #p<0.05 vs. only DOX-treated group.

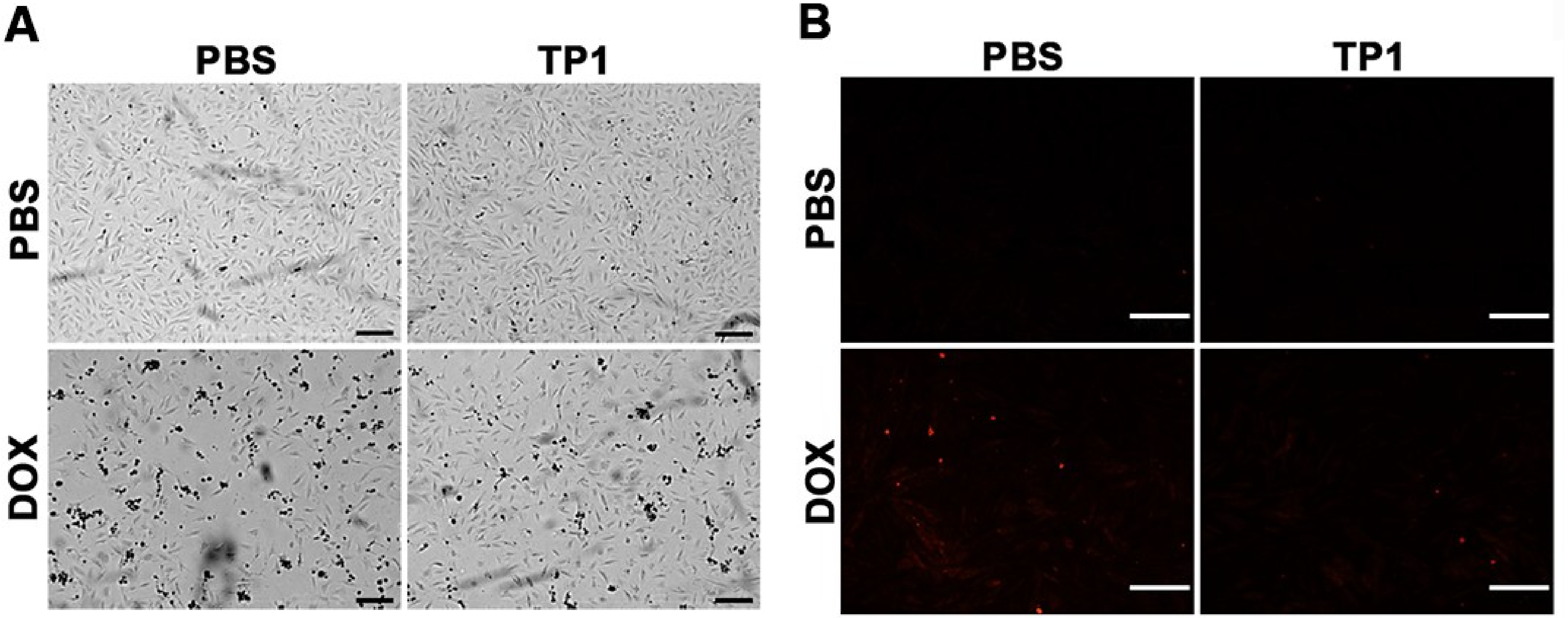

Figure S4: TP1 co-treatment pre-empts DOX-induced apoptosis in H9c2 cells. (A) Representative microscopic images showed altered cell morphology after DOX treatment. Typical morphological changes of H9c2 cells treated with DOX with or without TP1 after 24h. Scale bars, 200μm. (B) TP1 prevents DOX-induced apoptosis. Apoptosis cell populations were observed using PI staining of post-treated cells. Scale bars = 200μm.

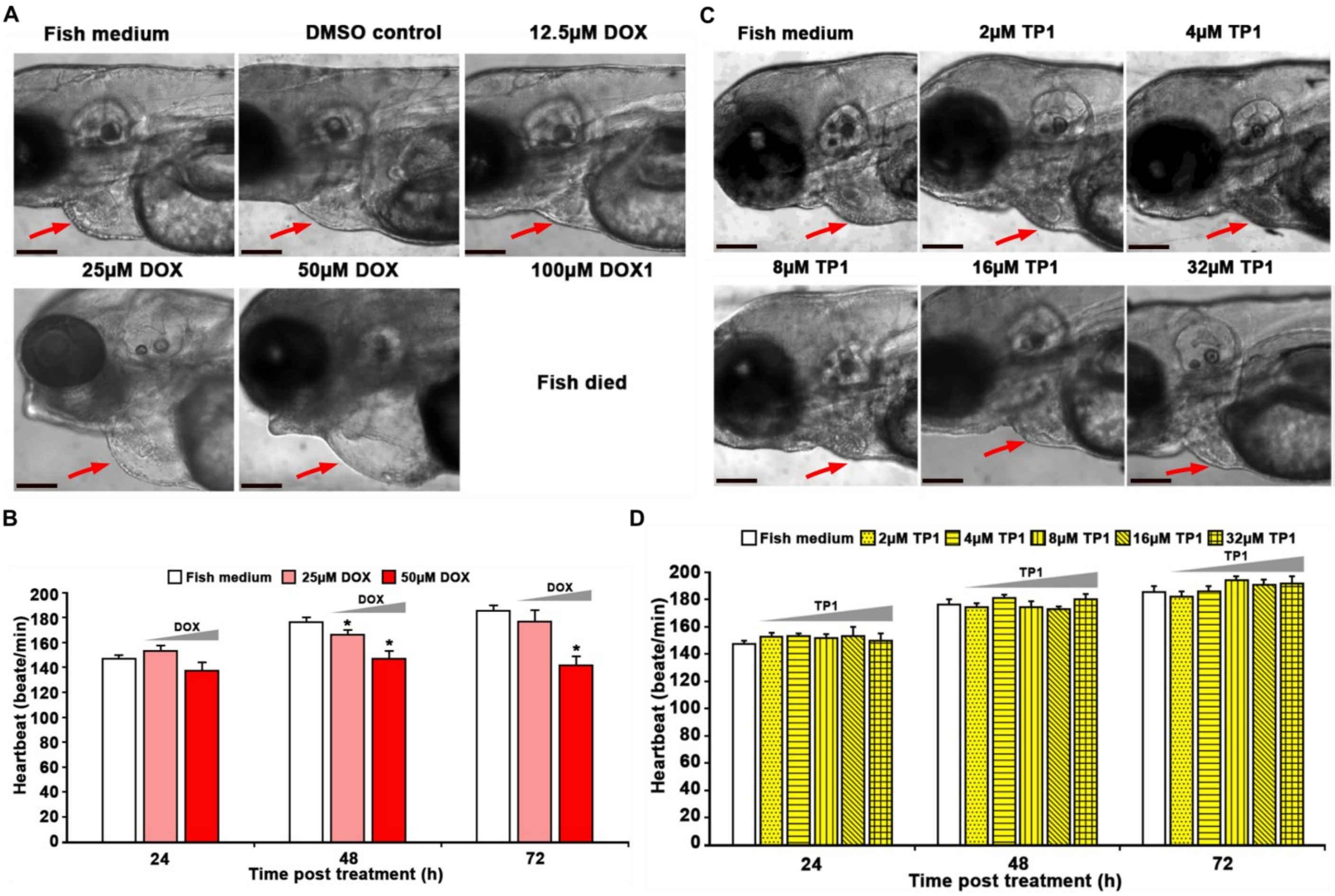

Figure S5: DOX-induced cardiotoxicity in zebrafish and Ginsentide TP1 is nontoxic. 24hpf zebrafish embryos were used to visualize and evaluate the cardiac functions. 24hpf embryos were treated with Sham control (Fish medium) or DOX for 72h. 15 individual embryos were used for each experimental group, and the treatment was performed in triplets. (A) DOX induces cardiotoxicity in zebrafish embryos—representative images of zebrafish embryos after 72 h DOX treatment. Red arrows indicate the cardiac sacs of zebrafish embryos and Scale bare=100μm. Enlarged cardiac sacs were detected in DOX-treated embryos, indicating DOX-induced pericardial edema. Fish died after 72h treatment with 100μM DOX. (B) DOX-induced cardiotoxicity reduces heartbeat rates. The heartbeat of the embryos was measured at 24h, 48h, and 72h time intervals after DOX treatment. Data was presented as mean (± SEM) of independent experimental triplicates (Analysis of variance (ANOVA) with Sidak's multiple comparisons test. *p<0.05 vs. the Sham control group). (C) Ginsentide TP1 is nontoxic to zebrafish embryos. 24hpf embryos were treated with TP1 for 72h. No significant visible abnormality was observed with the post-treated embryos. Red arrows indicate the cardiac sacs of zebrafish embryos and Scale bare=100μm. (D) TP1 has no significant impacts on cardiac function, and heartbeat rates remain normal after 72h post-treatment. The heartbeat of the embryos was measured at 24h, 48h, and 72h post TP1 treatment. Data was presented as mean (± SEM) of three independent experiments. (ANOVA with Sidak's multiple comparisons test. *p<0.05 vs. the Sham control group).

Group	Body Weight (g)				
	Day 1	Day 2	Day 3	Day 4	Day 5
**Control**	22.9±0.1	25.1±0.2	25.7±0.3	26.9±0.2	27.9±0.3
**Model**	23.5±0.2	25.0±0.3	23.3±0.2**	21.1±0.2**	19.5±0.3**
**RES 50mg/kg**	22.9±0.1	24.1±0.1##	22.8±0.3	20.3±0.3	18.9±0.3
**TP1 20mg/kg**	23.6±0.3	24.9±0.3	23.2±0.6	21.2±1.0	20.6±1.2

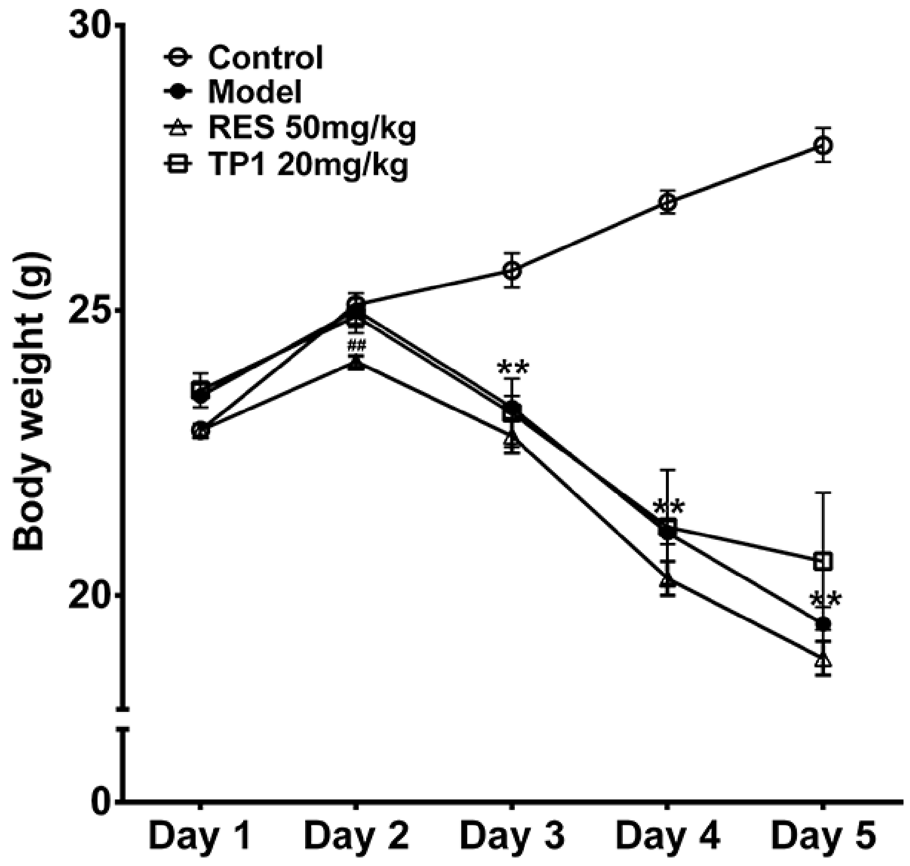

Figure S6: Pre-emptive administration of ginsentide TP1 has no significant impact on morbidity of DOX-induced acute myocardial injured mice. Male ICR mice were prophylactically treated with resveratrol (RES) (50 mg/kg) and TP1 (20 mg/kg), one dose per day for 5 days. DOX was injected intraperitoneally on day 2 to induce acute myocardial injury. The weight of the animals was monitored throughout the experiment to estimate the impact of oral or intraperitoneal administered RES or TP1 on morbidity in DOX-treated animals. Data represented as mean value with standard error (n=8, analysis of variance (ANOVA) with Sidak's multiple comparisons test. **P<0.01 vs. control, ## P<0.01 vs. model).

Full Blot images

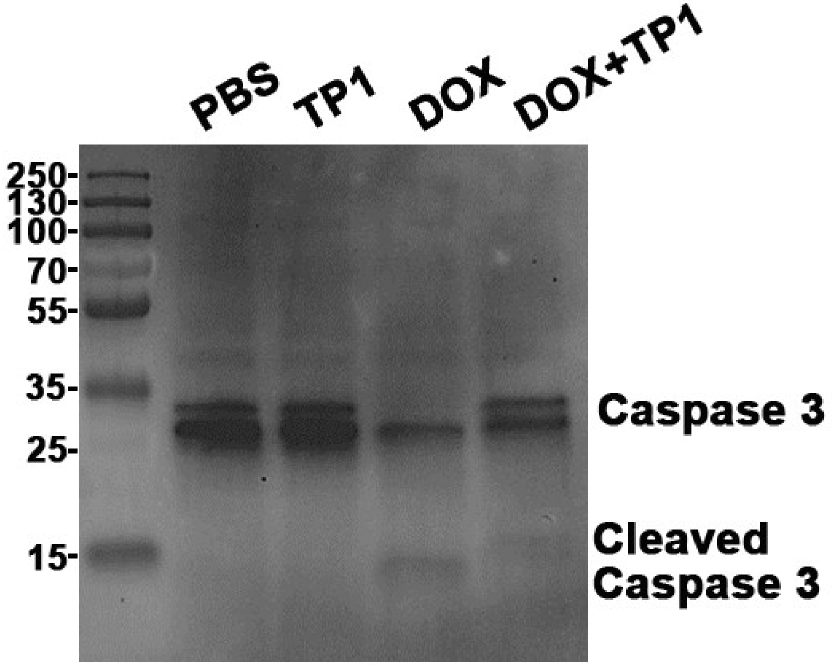

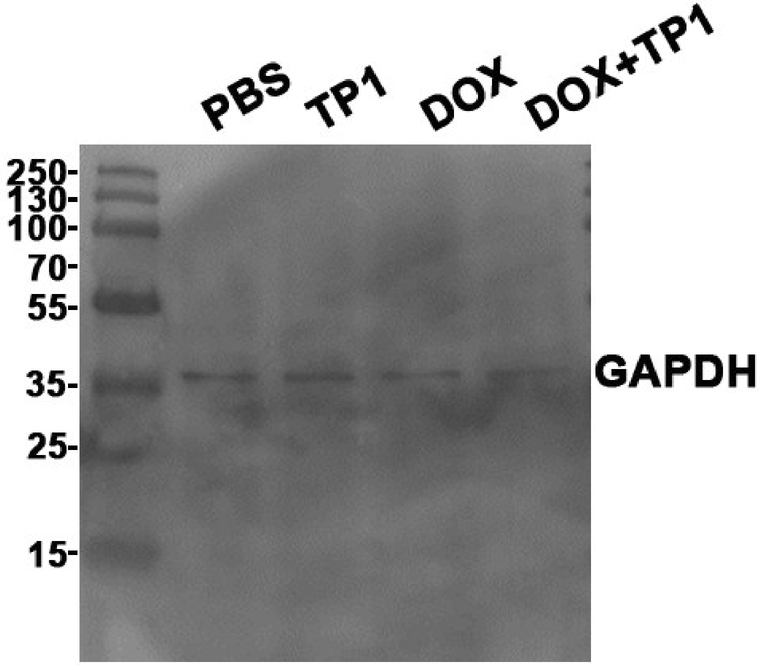

Video S1: Optical Mapping of Mock-treated zebrafish hearts. 24 hpf embryos were Sham control for 24h and subjected to measurement of cardiac parameters.

Video S2: Optical Mapping of DOX-treated zebrafish hearts shows slower heartbeats. 24 hpf embryos were treated with 50μM of DOX for 24h.

Video S3: Optical Mapping of 8μM TP1-cotreated zebrafish hearts shows restoration of heartbeats. 24 hpf embryos were cotreated with 50μM DOX and 8μM TP1 for 24h.

Video S4: Optical Mapping of 16μM TP1-cotreated zebrafish hearts reveals restoration of heartbeats to normal. 24 hpf embryos were cotreated with 50μM DOX and 16μM TP1 for 24h.

Video S5: Optical Mapping of Mock-treated Tg(cmlc2: gCaMP) Hearts. 24 hpf embryos were Sham control for 24h and subjected to measurement of cardiac parameters.

Video S6: Optical Mapping of DOX-treated Tg(cmlc2: gCaMP) hearts reveals reduced ventricular ejection fraction (EF). 24 hpf embryos were treated with 50μM for 24h and subjected to measurement of cardiac parameters.

Video S7: Optical Mapping of TP1-cotreated Tg(cmlc2: gCaMP) hearts reveals the restoration of ventricular ejection fraction (EF). 24 hpf embryos were cotreated with 50μM DOX and 16μM TP1 for 24h and subjected to measurement of cardiac parameters.
